# Can Meals Outside Homes Impact Sodium Intake?

**DOI:** 10.1093/cdn/nzaa091

**Published:** 2020-05-29

**Authors:** Ana Maria Pita Ruiz, Margareth Guimarães Lima, Lhais de Paula Barbosa Medina, Renata Luz Pinto, Marilisa Berti de Azevedo Barros, Antonio de Azevedo Barros Filho

**Affiliations:** Department of Collective Health, School of Medical Sciences, University of Campinas, Campinas 13083-970, SP, Brazil; Department of Collective Health, School of Medical Sciences, University of Campinas, Campinas 13083-970, SP, Brazil; Department of Collective Health, School of Medical Sciences, University of Campinas, Campinas 13083-970, SP, Brazil; Department of Collective Health, School of Medical Sciences, University of Campinas, Campinas 13083-970, SP, Brazil; Department of Collective Health, School of Medical Sciences, University of Campinas, Campinas 13083-970, SP, Brazil; Department of Collective Health, School of Medical Sciences, University of Campinas, Campinas 13083-970, SP, Brazil

**Keywords:** sodium, intake, meal, health survey, population

## Abstract

**Background:**

The WHO currently recommends a daily sodium intake of 2 g and has established the goal of a 30% reduction in mean salt intake by 2025.

**Objective:**

We sought to estimate sodium intake in study participants according to the locations of where they consumed meals and their demographic and socioeconomic characteristics and practices related to salt consumption.

**Methods:**

A population-based, cross-sectional study was conducted with a sample of 2574 individuals aged ≥10 y who answered the 2015 Campinas-Brazil Nutrition Survey. Mean sodium intake was estimated using a 24-h recall log and associations with the independent variables were tested using generalized regression analysis stratified by age group.

**Results:**

Sodium intake was higher in male participants as well as adolescents and adults who reported eating ≥1 meal outside the home (6.07% and 7.06% increase, respectively). Per meal, sodium was consumed more outside the home at breakfast, during an afternoon snack, and at dinner among adolescents. No significant differences were found in the analysis by type of meal among the adults and seniors.

**Conclusions:**

Sodium intake exceeded the WHO recommendation in all age groups analyzed. Having ≥1 meal outside the home was associated with greater sodium intake among adolescents and adults. Measures to regulate the food industry and dietary/nutritional education strategies targeting consumers are important to reducing the sodium intake of the population.

## Teaser

Sodium consumption is excessive in the entire population, and according to the findings of this study, is higher in adolescents and people who prefer to eat at least once a day outside the home.

## Background

In 2017, 32% of deaths in the world resulted from cardiovascular disease. Excessive sodium intake is one of the risk factors for the development of hypertension and, consequently, cardiovascular disease (1). The WHO currently recommends a daily sodium intake of 2 g (2) and has established the goal of a 30% reduction in mean salt intake by 2025 (3). Some countries of the American continent have an average daily sodium ingestion >2 g, ranging from 3.3 g in Canada to 4.7 g in Brazil (4, 5). Studies conducted in the Brazilian population show that >70% of individuals consume excessive quantities of sodium and >90% of adults and adolescents residing in urban areas consume quantities that surpass the recommended daily intake limit (4, 6, 7).

The results of the Intersal study carried out in 32 countries showed that an average sodium consumption of 2.3 g daily is related to increases of 4.5 and 2.3 mm Hg in systolic and diastolic blood pressure, respectively. In the high–sodium consumption group, increased blood pressure values also showed a positive association with age. On the other hand, these results were not observed in populations with low sodium consumption. These findings point out the importance of public policies to reduce sodium consumption in the general population (8).

Rapid urbanization, a busy lifestyle, and little time to prepare meals are factors that affect the dietary choices of the population (9, 10). Middle-income countries currently have a greater availability of ultra-processed foods with high calorie, fat, and sodium contents (11). Moreover, information on food labels is not clearly visible and uses language that is difficult to understand, hindering knowledge regarding the characteristics and quality of the products that consumers are acquiring (11, 12).

Nutritional guidelines found in the Dietary Guide for the Brazilian Population are based on the preferred consumption of natural or minimally processed foods, with meals preferably consumed at home and the ingestion of foods with low amounts of salt, sugar, and fat (13).

Studies indicate greater interest among consumers in knowing the nutritional content of the foods they acquire, giving preference to packaging with visible logotypes and healthy symbols rather than informative words (14). Moreover, greater nutritional knowledge is associated with lower consumption of unhealthy foods, such as those with a high sodium content (15–18). The excessive consumption of this micronutrient tends to be more common among men as well as segments of the population with a higher level of schooling (19). Sodium intake also decreases with age, demonstrating differentiation by age group (18, 19).

The location where meals are consumed is another important aspect to consider. Studies involving data from the Brazilian Family Budget Survey report a mean sodium intake of 4.7 g/d from foods available in the home (4). The authors suggest that this value may be even higher if one considers meals consumed outside the home (4, 7).

There are no population-based studies in Brazil evaluating sodium intake outside the home. The present population-based study fills this gap in the literature with an analysis of sodium intake according to the location where meals are consumed, the findings of which could contribute to the development of public health promotion and disease prevention policies addressing the consumption of this micronutrient. Therefore, the aim of the present study was to estimate sodium intake according to the location where meals are consumed and test associations with demographic and socioeconomic characteristics and practices related to salt consumption.

## Methods

A population-based, cross-sectional study was conducted using data from the 2015 Nutrition Survey conducted in the city of Campinas, state of São Paulo, Brazil. A total of 2641 interviews were conducted with individuals aged ≥10 y residing in urban areas of the city.

Two-stage, stratified, probabilistic, cluster sampling was performed for the survey. In the first stage, 70 census sectors were systematically selected, with probability proportional to size (given by the number of homes). The sectors were organized by mean income of the heads of households, and 14 sectors were selected from each of the 5 administrative districts of the city (20). The number of individuals in the sample was determined by considering a 50% proportion (to ensure maximum variability), a sampling error of 4 to 5 percentage points, and a design effect of 2. To obtain the desired sample, 3119, 1029, and 3157 homes were independently selected for interviews with adolescents, adults, and seniors, respectively, considering nonresponse rates of 27%, 22%, and 20%, respectively (21). More detailed information on the sampling process can be found in the sampling process manual available on the website of the faculty of medical sciences of the State University of Campinas (22).

The Campinas Nutrition Survey was part of the 2015 Campinas Health Survey addressing morbidities, the use of healthcare services, health-related behaviors, and social, economic, and demographic characteristics, etc. (20). A questionnaire was administered to the same sample of study articipants, addressing eating frequency, 24-h dietary recall, body perception, weight loss practices, self-rated diet quality, reading of food labels, presence of pesticides, and other information that enabled a nutritional and dietary analysis of the population.

Interviews of study participants were held on different days of the week (from Monday to Sunday). Most interviewees (89%) answered the food consumption questions between Monday and Friday. The 24-h dietary recall involved the use of the “multiple-pass method” proposed by the USDA (23).

The 24-h recall was administered with the aid of a photographic manual and recorded in household measurement units, which were subsequently quantified in grams and milliliters by trained staff who used tables of household units to standardize the amount of sodium used in food preparation and added at the table during meals (23–25). Data were imported to the Nutrition Data System for Research (NDS-R) version 2015 (Nutrition Coordinating Center), Additional data, such as information about traditional Brazilian culinary preparations and the amount of salt added at the table (20). The data from the questionnaire of the Campinas Nutrition Survey were entered twice into the database using blinded data entry and the EpiData software, version 3.1, and then checked for consistency (20).

## Variables

The dependent variable was mean daily sodium intake in grams. The main independent variable was the location of food consumption. The data for this variable were obtained from an open-ended question “places of food consumption in the last 24 hours.” The interviewees answered by providing information regarding all the places in which they had eaten in the previous 24 h. Different results were obtained, and the main answers were the following: home, work, school, restaurant, friend's house, at a party, travel, and store, among others. Later, the answers were categorized into 2 settings: (home and other than home). The other independent variables are listed below.

### Demographic and socioeconomic characteristics

Data for demographic and socioeconomic characteristics of the study participants included sex (male, female), race (white, black/brown), age group (10–19, 20–59, or ≥60 y), years of schooling, and family income per capita. Years of schooling were recorded as head of household years of schooling for adolescents (0–8, 9–11, or ≥12 y of study) and years of schooling for adults (0–8, 9–11, or ≥12 y of study) and seniors (0–3, 4–8, or ≥9 years of study). Family income per capita was recorded using the Brazilian monthly minimum wage (BMMW) as reference (<BMMW, 1 to 2 times the BMMW, or >2 times the BMMW for adolescents and <BMMW, >1 to ≤ 3 times the BMMW, or >3 times the BMMW for adults and seniors).

### Practices related to salt intake

Data for practices related to salt intake included concern with salt intake (yes, no), self-rated salt intake (inadequate, adequate), meal (breakfast, lunch, afternoon snack, dinner), and reading of salt content on food labels (yes, no).

### Statistical analysis

Mean sodium intake according to demographic and socioeconomic characteristics and intake related to diet were calculated and associations were tested using a linear regression model corrected using the jackknife method (26, 27). A hierarchical multivariate model was used, with the incorporation of variables performed with the stepwise backward method (28, 29). The following 3 steps were performed for analysis: *1*) a bivariate analysis was performed for all variables of the study, *2*) a multivariate analysis was performed in which the variables with a *P* value of <0.20 for stage 1 remained, and *3*) the variables with a *P* value of <0.05 were used in the last multivariate stage. We adjusted the results by the total energy value. Residual analyses were performed for each model and they showed favorable results. The analyses were stratified by age group, with the level of significance set to 5%, and performed and programmed with the aid of STATA 14.0. The *svy* command was used, which considers the weights of the research design, the weight of the nonresponse, and the weight of the stratification.

### Ethical considerations

This study received approval from the Human Research Ethics Committee of the State University of Campinas (certificate no. 22425019.3.0000.5404) and the National Ethics Committee (CEP/CONEP system).

## Results

The mean sodium intake in the sample population was 3.3 g/d (3.73 g/d for males and 2.87 g/d for females, *P* <0.0001); thus, females consumed 0.86 g/d less sodium than males. After adjusting for the total energy value of the diet, the association between sodium intake and sex remained significant (*R*: −0.24; *P* <0.0001). Sodium intake was higher among adolescents (3.52 g/d) than adults (3.34 g/d) and seniors (3.01 g/d). After adjusting for the total energy value of the diet, the association between sodium intake and age remained significant (*R*: −0.05; *P* = 0.025) and (*R*: −0.20; *P = *0.0001) (data not presented in tables).

For the adolescent study participants, the bivariate analysis showed greater sodium intake in males (**Table 1**). In the multivariate model, the meal consumption location was associated with mean sodium intake even after the adjustment for sex (*R*: 0.19; *P = *0.012). Adolescents who consumed ≥1 meal outside the home had an excess of 0.19 g/d of sodium, corresponding to a 6.07% increase compared with sodium in adolescents who had meals exclusively at home.

**TABLE 1:** Mean sodium intake and regression coefficients of associations with demographic and socioeconomic characteristics and practices related to salt consumption in an adolescent population in Campinas, San Paulo, Brazil.^1^

	Bivariate model (g/d)				Multivariate model (g/d)					
	*n*	Mean^2^	95% CI	*P* value	Stage 1		Stage 2		Stage3	
Variables					*R* ^3^	*P* value	*R* ^3^	*P* value	*R* ^4^	*P* value
Sex										
Male	458	3.89	3.71, 4.06							
Female	443	3.11	2.96, 3.25	<0.0001	−0.79	<0.0001	−0.79	<0.0001	−0.40	<0.0001
Race/skin color										
White	492	3.43	3.25, 3.60							
Black/brown	391	3.59	3.41, 3.78	0.18	0.14	0.25	—	—	—	—
Schooling of head of family										
0–8 y	475	3.48	3.33, 3.63							
9–11 y	266	3.66	3.46, 3.86	0.87	—	—	—	—	—	—
≥12 y	147	3.29	2.99, 3.57	0.49	—	—	—	—	—	—
Income per capita										
≤BMMW ^a^	545	3.5	3.35, 3.66							
1–2 x BMMW	275	3.55	3.34, 3.77	0.70	—	—	—	—	—	—
>2 x BMMW	79	3.3	2.87, 3.72	0.34	—	—	—	—	—	—
Location of meals										
At home	575	3.44	3.29, 3.59							
Outside of home	219	3.50	3.25, 3.75	0.059	—	—	0.19	0.012	0.12	0.038
Reads sodium content on labels										
No	250	3.61	3.42, 3.80							
Yes	30	3.42	2.44, 4.44	0.72	—	—	—	—	—	—
Concerned about salt intake										
No	590	3.54	3.39, 3.68							
Yes	310	3.43	3.21, 3.65	0.39	—	—	—	—	—	—
Self-rated salt intake										
Inadequate	132	3.57	3.30, 3.83							
Adequate	765	3.49	3.34, 3.63	0.59	—	—	—	—	—	—

1 BMMW, Brazilian monthly minimum wage.

2 Mean sodium intake (in grams) based on a 1-d 24-h dietary recall.

3 Regression coefficient.

4 Regression coefficient adjusted for total energy.

Among the adult study participants, sodium intake was higher in males (**Table 2**). The multivariate model showed that the meal consumption location remained associated with sodium intake even after the adjustment for sex (*R*: 0.22; *P* = 0.005). Adults who consumed ≥1 meal outside the home had an excess of 0.22 g/d of sodium, corresponding to a 7.06% increase compared with that in adults who consumed meals exclusively at home.

**TABLE 2:** Mean sodium intake and regression coefficients of associations with demographic and socioeconomic characteristics and practices related to salt consumption in an adult population in Campinas, San Paulo, Brazil.^1^

	Bivariate model (g/d)				Multivariate model (g/d)					
					Stage 1		Stage 2		Stage 3	
Variables	*n*	Mean^2^	CI	*P* value	*R* ^3^	*P* value	*R* ^3^	*P* value	*R* ^4^	*P* value
Sex										
Male	372	3.79	3.56, 4.10							
Female	480	2.88	2.75, 3.00	<0.0001	−0.91	<0.0001	−0.90	<0.0001	−0.70	<0.0001
Race/skin color										
White	532	3.24	3.07, 3.41							
Black/brown	301	3.40	3.22, 3.59	0.20	—	—	—	—	—	—
Schooling										
0–8	326	3.25	3.05, 3.45							
9–11 y	300	3.40	3.20, 3.60	0.30	—	—	—	—	—	—
≥12 y	225	3.25	3.00, 3.49	0.98	—	—	—	—	—	—
Income per capita										
≤BMMW	337	3.27	3.00, 3.53							
1–3 x BMMW	413	3.30	3.14, 3.52	0.71	—	—	—	—	—	—
>3 x BMMW	102	3.31	3.01, 3.61	0.84	—	—	—	—	—	—
Location of meals										
At home	483	3.12	2.93, 3.33							
Outside of home	197	3.49	3.25, 3.73	0.002	—	—	0.22	0.005	0.17	0.010
Reads sodium content on labels										
No	354	3.31	3.07, 3.55							
Yes	87	3.39	3.02, 3.76	0.75	—	—	—	—	—	—
Concerned about salt intake										
No	327	3.37	3.16, 3.57							
Yes	525	3.23	3.14, 3.42	0.36	—	—	—	—	—	—
Self-rated salt intake										
Inadequate	114	3.51	3.21, 3.81							
Adequate	737	3.27	3.13, 3.41	0.114	−0.22	0.10	—	—	—	—

1 BMMW: Brazilian monthly minimum wage.

2 Mean sodium intake (in grams) based on one-day 24-h dietary recall.

3 Regression coefficient.

4 Regression coefficient adjusted by total energy.

Among the senior study participants, sodium intake was higher among males but was not associated with the location where meals were consumed (**Table 3**).

**TABLE 3:** Mean sodium intake and regression coefficients of associations with demographic and socioeconomic characteristics and practices related to salt consumption in a senior population in Campinas, San Paulo, Brazil.^1^

	Bivariate model (g/d)				Multivariate model (g/d)					
	Stage 1						Stage 2		Stage 3	
Variables	*n*	Mean^2^	CI	*P* value	*R* ^3^	*P* value	*R* ^3^	*P* value	*R* ^4^	*P* value
Sex										
Male	325	3.39	3.22, 3.55							
Female	496	2.63	2.37, 2.88	<0.0001	−0,74	<0.0001	−0,74	<0.0001	−0.45	0.001
Race/skin color										
White	532	3.04	2.80, 3.23							
Black/brown	301	2.85	2.64, 3.06	0.25	—	—	—	—	—	—
Schooling										
0–3 y	295	2.65	2.47, 2.81							
4–8 y	350	3.00	2.85, 3.16	0.002	0.25	0.032	—	—	—	—
≥8 y	172	3.37	2.81, 3.93	0.015	0.64	0.11	—	—	—	—
Income per capita										
≤BMMW	259	2.70	2.54, 2.85							
1–3 x BMMW	447	3.03	2.74, 3.32	0.018	0.16	0.17	—	—	—	—
>3 x BMMW	113	3.07	2.78, 3.35	0.016	−0.02	0.92	—	—	—	—
Location of meals										
At home	717	2.89	2.70, 3.08							
Outside of home	69	3.17	2.83, 3.52	0.067	—	—	—	—	—	—
Reads sodium content on labels										
No	358	2.93	2.76, 3.10							
Yes	63	3.00	2.61, 3.40	0.71	—	—	—	—	—	—
Concerned about salt intake										
No	218	2.93	2.72, 3.13							
Yes	599	2.93	2.71, 3.15	0.80	—	—	—	—	—	—
Self-rated salt intake										
Inadequate	61	3.15	2.76, 3.55							
Adequate	755	2.91	2.32, 3.09	0.71	—	—	—	—	—	—

1 BMMW, Brazilian monthly minimum wage

2 Mean sodium intake (in grams) based on 1-d 24-h dietary recall.

3 Regression coefficient.

4 Regression coefficient adjusted by total energy.

Among the adolescent study participants, 15.97% of meals were consumed outside the home: 6.6% at school and 9.37% in other places, such as at restaurants (3.40%) and work (3.39%). Among the adults, the prevalence of ≥1 meal outside the home was 20.3%; the highest location frequencies were at work (12.63%), restaurants (5.13%), and other places (2.54%). Among the seniors, the prevalence of ≥1 meal outside the home was 4.64%; the highest frequencies were at work (2.03%) and restaurants (1.43%) (data not presented in tables).

The distribution of meals consumed outside the home was similar among all age groups, with afternoon snack the most frequent meal consumed outside the home (28.9%), followed by lunch (23.9%), dinner (23.3%), and breakfast (22.7%). Regarding consumption outside the home, 24.34% of adolescents, 23.12% of adults, and 8.45% of seniors consumed food outside the home once a day. Also, 11.58%, 20.19%, and 3.79% of adolescents, adults and seniors, respectively, consumed food outside the home ≥2 times during the day. (data not presented in tables).

**Table 4** shows a significant increase in sodium consumption among adolescents who ate breakfast (*R*: 0.16; *P* = 0.032), an afternoon snack (*R*: 0.09; *P* = 0.002), and dinner (*R*: 0.30; *P* = 0.029) outside the home. No significant differences were found in the analysis by type of meal among the adults and seniors.

**TABLE 4:** Mean sodium intake (in grams) and regression coefficients of associations with location of consumption and meals/person stratified by age group. Campinas, SP, Brazil.

	Mean (g)						
Meal	*n*	At home^1^	*n*	Outside home^2^	*R* ^3^	CI	*P* value^4^
Adolescents							
Breakfast	700	0.42	42	0.58	0.16	0.01, 0.30	0.032
Lunch	701	1.51	157	1.47	−0.03	−0.20, 0.12	0.62
Afternoon snack	770	0.31	289	0.43	0.09	0.04, 0.50	0.002
Dinner	791	1.30	69	1.55	0.30	0.03, 0.57	0.029
Adults							
Breakfast	727	0.36	81	0.44	0.09	0.03, 0.21	0.12
Lunch	547	1.49	273	1.50	0.05	−0.09, 0.19	0.49
Afternoon snack	648	0.22	242	1.98	−0.02	−0.09, 0.05	0.51
Dinner	726	1.32	70	1.49	0.19	0.08, 0.46	0.15
Seniors							
Breakfast	792	0.32	18	0.31	0.01	−0.16, 0.18	0.98
Lunch	746	1.31	63	1.34	0.08	−0.10, 0.27	0.38
Afternoon snack	987	0.22	67	0.20	0.17	−0.03, 0.34	0.09
Dinner	760	1.10	9	0.95	−0.15	−0.44, 0.14	0.31

1 Meals/person consumed at home.

2 Meals/person consumed outside of home.

3 Adjusted for sex.

4 Significance of difference in sodium intake at meals consumed at home and outside of home.

## Discussion

Mean sodium intake was higher than that recommended by the WHO in all age groups of the present sample. The findings also show greater mean sodium intake among males and adolescents as well as higher intake among individuals who consumed ≥1 meal outside the home, except among the seniors. In the stratification by type of meal, sodium intake was higher at breakfast, during the afternoon snack, and at dinner among adolescents who consumed ≥1 of these meals outside the home compared with those who consumed meals exclusively at home.

The greater sodium intake among males and adolescents is in agreement with data obtained in a study conducted in 5 sentinel countries of the Americas, which demonstrated substantially lower sodium intake among women and older individuals (18). As women seek healthcare services more frequently than men (30), it is possible that women are also better informed regarding the consumption of this micronutrient. Moreover, the responsibility for the dietary care of the family has historically been in the hands of women. However, the increased inclusion of women in the job market may lead to an increase in the consumption of meals outside the home (31).

Regarding the association between mean sodium intake and the location where meals are consumed, the Dietary Guide for the Brazilian Population states that it is preferable for meals to be made and consumed at home with plates that make up part of the culture and culinary tradition of families, with the purpose of restoring the connection to foods and enabling knowledge on the origin and ingredients of each plate (13, 32). Moreover, when meals need to be consumed outside the home, preference should be given to restaurants offering a buffet, which enables choices based on individual needs and facilitates the control of the addition of spices, sauces, and salt. However, in a study conducted in the city of Chapecó (state of Santa Catarina, Brazil) found that the mean sodium intake at lunch in restaurants that offer a buffet was 1.74 g, 52.25% of which was from processed foods (33). This finding shows that a single meal consumed in a restaurant can account for 80% of the recommended daily sodium intake.

The present findings draw attention to the greater sodium intake among adolescents who ate breakfast and/or an afternoon snack and/or dinner outside the home. This segment of the population consumed the largest part of these meals at school and work. The diet of adolescents has been characterized by the regular consumption of foods that are markers of an unhealthy diet (34, 35). Moreover, advertising from the food industry does not prioritize nutritional content, but rather appeals to feelings of belonging, satisfaction, and pleasure, persuading consumers to ingest foods with high calorie, fat, sugar, and/or salt content (36). Thus, health promotion actions directed at youths are needed (34). The Brazilian National School Meal Program has restricted the consumption of ready-to-eat, canned, and processed foods and has established the following limit regarding the sodium content per meal: schools are only permitted to offer a maximum of 0.40 g of this micronutrient per meal (37, 38). It is important to see schools as a place for the promotion of healthy eating (38, 39). Beginning in 2018, dietary and nutritional education activities have been included in basic education (40). Moreover, dietary and nutritional education activities directed at the entire family are important, with the stimulation of the creation of school vegetable gardens and the strengthening of cooking skills to generate a connection to foods from production through to consumption (38, 39).

Among adolescents, the greater sodium intake at dinner may occur in restaurants, fast food diners, bakeries, supermarkets, etc. (41). A study conducted at 6 restaurant chains in the United States showed that a reduction in the sodium content of food served was accepted by consumers and, given the frequency with which they eat outside the home, such reductions are capable of improving the diet of restaurant clients (42). Every strategy for reducing the sodium content of meals is significant and impactful, especially regarding meals consumed outside the home, which were found in the present investigation to be significantly higher in sodium content.

In Brazil, the 2012–2015 National Health Plan and the Strategic Plan for Combatting Chronic Diseases (2011–2022) outline strategies for surveillance, monitoring, health promotion, combatting risk factors, and strengthening healthcare systems, together with the encouragement of healthy eating through education, information, and the reformulation of the sodium content of processed foods (43). In 2011, the Brazilian Health Ministry selected priority food categories that contribute >90% of sodium intake through processed foods and established voluntary adherence goals with the food industry to reduce the sodium content in its products (44, 45). However, these actions have had little impact on the mean sodium intake of the population. The mean reduction in 2016 was 1.5%. After the application of a 25% reduction in sodium in processed foods in 2017, the mean estimated reduction was 6.3% in the population (46).

Considering the mean sodium intake in the population of Campinas (3.30 g/d), a reduction of >1.00 g/d is needed to meet the maximum limit recommended by the WHO. The results of the present study also reveal that, although sodium content at home surpasses the recommendation, the sodium intake of the population of Campinas could be reduced by 6.07% among adolescents and 7.06% among adults by restricting meals to the home. A study conducted in New Zealand found that a 36% reduction in the sodium content of processed foods together with a 40% reduction in the sodium content of foods consumed outside the home would reduce the mean sodium intake of the population to within the limit established by the WHO (47).

The present study has limitations that should be considered. The 24-h dietary recall limited to a single day may not necessarily demonstrate habitual consumption and does not consider intraindividual variability, and a memory bias can be found since there is a dependence on the recent memory of the studied subjects (48). The best method for evaluating sodium intake is 24-h urinary excretion (49). However, this is difficult to do in a population-based study. This study also has strengths that should be pointed out. The evaluation of the effective sodium intake according to demographic and socioeconomic characteristics and practices related to salt consumption provides updated information on the sodium intake of the population. The intake of this micronutrient was also evaluated considering the location where meals are consumed, which fills a gap in the literature on sodium intake during meals consumed outside the home. Other strengths of this study were the fact that the sample was representative of the population of a large city and adjustments were made for potential confounding factors.

## Conclusion

Mean sodium intake exceeded the limit established by the WHO in all age groups of the present sample. Having ≥1 meal outside the home was associated with greater sodium intake among adolescents and adults. Measures to regulate the food industry and dietary and nutritional education strategies targeting consumers are important in reducing the sodium intake of the population.

## Notes

Supported by the Fundação de Amparo à Pesquisa do Estado de São Paulo [FAPESP (State of Sao Paulo Research Assistance Foundation)], which funded the ISACamp 2015 (grant 2012/23324-3) and ISACamp-Nutri 2015 (grant 2013/16808-7) studies; the Coordenação de Aperfeiçoamento de Pessoal de Nível Superior [CAPES ([Coordination for the Advancement of Higher Education Personnel)], which funded the master's degree grant of Ana Maria Pita Ruiz; and Scientific Development (CNPq) funded the productivity scholarship granted to MBdAB (grant 309073/2015-4).

Author disclosures: The authors report no conflicts of interest.

Abbreviations used: 24-h recall, 24-hour dietary recall; BMMW, Brazilian monthly minimum wage; NDS-R, Nutrition Data System for Research.
